# Widespread Occurrence of Chemical Residues in Beehive Matrices from Apiaries Located in Different Landscapes of Western France

**DOI:** 10.1371/journal.pone.0067007

**Published:** 2013-06-17

**Authors:** Olivier Lambert, Mélanie Piroux, Sophie Puyo, Chantal Thorin, Monique L'Hostis, Laure Wiest, Audrey Buleté, Frédéric Delbac, Hervé Pouliquen

**Affiliations:** 1 LUNAM Université, Oniris, Ecole Nationale Vétérinaire, Agroalimentaire et de l'Alimentation Nantes-Atlantique, Plateforme Environnementale Vétérinaire, Centre Vétérinaire de la Faune Sauvage et des Ecosystèmes des Pays de la Loire (CVFSE), Nantes, France; 2 LUNAM Université, Oniris, Ecole Nationale Vétérinaire, Agroalimentaire et de l'Alimentation Nantes-Atlantique, Unité de Physiopathologie Animale et Pharmacologie Fonctionnelle, Nantes, France; 3 Université de Lyon, Institut des Sciences Analytiques, Département Service Central d'Analyse, UMR 5280 CNRS, Université de Lyon1, ENS-Lyon, Villeurbanne, France; 4 Clermont Université, Université Blaise Pascal, Laboratoire Microorganismes: Génome et Environnement, BP 10448, Clermont-Ferrand, France; 5 CNRS, UMR 6023, LMGE, Aubière, France; French National Institute for Agricultural Research (INRA), France

## Abstract

**Background:**

The honey bee, *Apis mellifera*, is frequently used as a sentinel to monitor environmental pollution. In parallel, general weakening and unprecedented colony losses have been reported in Europe and the USA, and many factors are suspected to play a central role in these problems, including infection by pathogens, nutritional stress and pesticide poisoning. Honey bee, honey and pollen samples collected from eighteen apiaries of western France from four different landscape contexts during four different periods in 2008 and in 2009 were analyzed to evaluate the presence of pesticides and veterinary drug residues.

**Methodology/Findings:**

A multi-residue analysis of 80 compounds was performed using a modified QuEChERS method, followed by GC-ToF and LC−MS/MS. The analysis revealed that 95.7%, 72.3% and 58.6% of the honey, honey bee and pollen samples, respectively, were contaminated by at least one compound. The frequency of detection was higher in the honey samples (n = 28) than in the pollen (n = 23) or honey bee (n = 20) samples, but the highest concentrations were found in pollen. Although most compounds were rarely found, some of the contaminants reached high concentrations that might lead to adverse effects on bee health. The three most frequent residues were the widely used fungicide carbendazim and two acaricides, amitraz and coumaphos, that are used by beekeepers to control *Varroa destructor*. Apiaries in rural-cultivated landscapes were more contaminated than those in other landscape contexts, but the differences were not significant. The contamination of the different matrices was shown to be higher in early spring than in all other periods.

**Conclusions/Significance:**

Honey bees, honeys and pollens are appropriate sentinels for monitoring pesticide and veterinary drug environmental pollution. This study revealed the widespread occurrence of multiple residues in beehive matrices and suggests a potential issue with the effects of these residues alone or in combination on honey bee health.

## Introduction

Since the middle of the twentieth century, profound changes have occurred and damaged the ecological balance. Industrialization, growing urbanization, transportation and agricultural practices have led to overall ecosystem contamination and to major modifications in landscape structure and composition. These changes have had adverse effects on biodiversity, causing physiological and behavioral damage to living organisms and altering organism habitats and the quality and/or the quantity of food resources [1]–[5]. The consequences have been disastrous, particularly as supplementary stressors, such as infectious agents or invasive species, may be added [6]–[9].

Bees are at the center of this issue. First, although honey bee populations are globally increasing throughout the world, unprecedented colony losses have been reported in Europe and North America over the last decade, and the number of hives that must be replaced each year has drastically increased [10]–[11]. Multiple causes are suspected including (i) climate change; (ii) reduction of floral diversity and quality resources in relation to monocultural practices and fragmentation of natural habitats; (iii) infection by pathogens, including viruses, bacteria, fungi and parasites [12]–[15]; and (iv), poisoning by chemical compounds, including pesticides [16]–[17]. Even if each individual cause may have a real impact on honey bee health, no factor has emerged as the definitive and single stressor responsible for this decline. Many authors actually suggest that pesticides are not involved [12] because most field studies have demonstrated that pesticides have not been found at levels that would be harmful to bees. Thus, a combination of biological, chemical and physical stressors would be the most probable explanation for extensive colony losses. In particular, recent studies have reported that parasite-insecticide interactions can synergistically and negatively affect honey bee survival [18]–[22].

Second, honey bee and other beehive matrices are recognized as appropriate sentinels for monitoring anthropogenic contamination in the environment [23]–[24]. Honey bees are exposed to atmospheric pollutants during their foraging activities, their hairy bodies easily hold residues, and they may be exposed to contaminants *via* contaminated food resources such as nectar, pollen or water. Therefore, many studies have used honey bees, pollen or honey as relevant samples to assess the levels of heavy metals [25]−[26] and polycyclic aromatic hydrocarbons [27] in both wild and anthropogenic areas, but pesticides are also of concern in agricultural areas [28]–[31].

In this context, the Wildlife and Ecosystems Veterinary Center of Pays de la Loire (CVFSE/Oniris) conducted a program concerning the use of honey bees (*Apis mellifera* L.), honey and pollen for monitoring lead [32], polycyclic aromatic hydrocarbons [33] and pesticide environmental pollution in Pays de la Loire (western France). The aim of the present study was to investigate the contamination of 18 apiaries by pesticide residues through analyses of 3 different matrices over 2 years (2008 and 2009). The sampled apiaries were located in four different landscapes susceptible to various contamination levels due to different uses of pesticides and veterinary drugs (gardening, agricultural, herd breeding or apicultural practices). To our knowledge, this study is the first to compare the contamination of 3 matrices in 4 different landscape contexts. The temporal distribution of the pesticide and veterinary drug concentrations and the choice of the most relevant matrices for monitoring environmental pesticide and veterinary drug contamination are discussed.

## Materials and Methods

### Study sites

**Figure 1:**
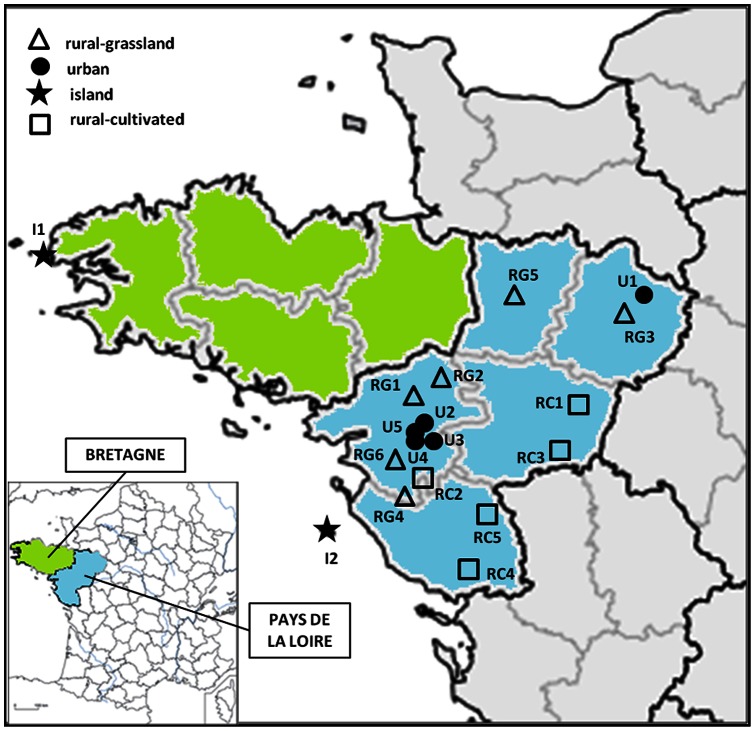
Location of the 18 surveyed apiaries. The apiaries are located in four different landscape contexts (rural-grassland landscapes: RG1 to RG6, rural-cultivated landscapes: RC1 to RC5, urban landscapes: U1 to U5, islands: I1 and I2) from two regions of western France (Bretagne in green and Pays de la Loire in blue).

Apicultural matrices were collected from 18 apiaries located in four different landscapes from western France (Bretagne and Pays de la Loire) (**Fig. 1**). Two apiaries were located on small islands (Isle of Ouessant, I1, and Isle of Yeu, I2) that are free from high levels of anthropogenic activities. These islands were selected to represent landscapes with low levels of pesticides. Six apiaries (RG1 to RG6) were located in a rural-grassland landscape characterized by high length of hedges and numerous grassland plots. Five apiaries (RC1 to RC5) were located in a rural-cultivated landscape characterized by large plots of crops (permanent, oil seed, grain crops, and market gardening) and a low hedgerow network. The pesticide display in these 11 rural-sites is reflective of agricultural practices and veterinary treatments of farm animals. Finally, five apiaries (U1 to U5) were located in an urban landscape characterized by large urban areas and some rural areas. The observed pesticides in these apiaries are reflective of leisure gardening, and a small number of these pesticides emanate from agricultural treatments.

### Sample collection

Three different biological matrices (foraging honey bees, trap pollen and honey) were collected from eight colonies randomly selected at each apiary. The samples were always collected from the same eight colonies during the survey. Otherwise, the number of hives sampled was kept similar by replacement of each dead colony. For the final assessment, this replacement was not subjected to a special statistical treatment. The apiaries were visited four times in both 2008 (periods AM8, JJ8, JA8 and SO8) and 2009 (periods AM9, JJ9, JA9 and SO9). In terms of seasons, the apiaries were visited in spring (late April-early May, periods AM8 and AM9), at the beginning of summer (late June-early July, periods JJ8 and JJ9), in summer (late July-early August, periods JA8 and JA9), and at the beginning of autumn (late September-early October, periods SO8 and SO9). During a single period, all samples were collected when possible within 10 days to minimize variations in climatic factors, flowering and pesticide treatments. The owners of the studied apiaries were present for each sampling, and their names are being kept confidential.

The honey bees were directly collected from the hive's flight board with a hand-held vacuum cleaner. The pollens were collected in pollen traps installed by beekeepers three days before the sampling. As foraging activities depend on meteorological conditions, some pollen samples were missing due to low temperatures or bad weather, especially at the beginning of autumn. Honey samples were collected from several honeycombs with a cutter or with a punch. Uncapped honeycombs were chosen (when possible in beehive rises) to collect fresh honey. In an apiary, each colony displays specific foraging activities that may not be representative of the whole apiary. However, for the purpose of this study, *i.e*., the use of the apicultural matrices as sentinels for monitoring the contamination by pesticides and veterinary drugs around or in each apiary, samples of honey bees, honey and pollen collected in the hives of the same apiary and at the same period were pooled. These field-collected pools were immediately placed on ice after sampling and then stored at −20°C until analysis. In total, 141 honey bee samples, 141 honey samples and 128 pollen samples were collected.

### Sample preparation, analysis and method performance

A multi-residue analysis was developed to identify and quantify 80 pesticides (gardening and agricultural) and veterinary drugs in the three beehive matrices. The 80 compounds covered 21 families of contaminants and corresponded to the majority of pesticides used for plant protection and some veterinary drugs used for treatments of farm animals or in apicultural practices to control the parasitic mite *Varroa destructor* (**Table 1**). The method consisted of a single extraction, based on a modified “QuEChERS method” (“Quick Easy Cheap Effective Rugged Safe method”), followed by gas chromatography coupled with time-of-flight mass spectrometry (GC-ToF) and liquid chromatography coupled with tandem mass spectrometry (LC−MS/MS) as previously described [34]. The combination of the “QuEChERS method” with the sensitive GC-ToF and LC−MS/MS analytical techniques enabled the detection of pesticide concentrations as low as 10 ng/g in honey bee, honey and pollen samples. The limit of detection (LOD) and limit of quantification (LOQ) of each chemical compound are given in **Table 1**.

**Table 1:** Method, limits of detection and limits of quantification for the 80 compounds analyzed for beehive matrices from western France honey bee colonies.

Compound	Method^1^1	Class^2^	Effect^3^	Honey bees		Honey		Pollen	
				LOD^4^	LOQ^5^	LOD^4^	LOQ^5^	LOD^4^	LOQ^5^
4,4′-dichlorobenzophenone	GC	OC	A	3.6	9.0	3.6	17.9	3.1	11.2
Abamectin	LC	AVER	I	10.2	20.4	10.2	30.6	nd	nd
Aldrin	GC	OH	I	4.5	22.3	0.2	4.5	11.1	13.9
Amitraz I	LC	FORM	I	18.5	27.8	10.0	37.0	46.3	69.4
Amitraz II	LC	FORM	I	4.3	10.8	0.3	4.3	8.1	17.3
Benalaxyl	GC	PHENA	F	5.7	28.4	5.7	14.2	21.3	42.7
Bifenthrin	GC	PYRE	I	1.3	5.1	3.3	12.9	4.5	19.3
Bitertanol	GC	TRIA	F	1.1	4.4	11.0	16.5	3.9	16.5
Bromopropylate	GC	CARBI	A	0.2	3.9	0.3	3.9	1.0	14.5
Bupirimate	GC	PYRI	F	5.7	14.2	5.7	14.2	2.8	21.4
Buprofezine	GC	THIAD	I	23.9	71.8	23.9	35.9	29.9	59.9
Cadusaphos	GC	OP	N	1.0	8.9	3.6	8.9	8.9	22.3
Carbaryl	LC	CARB	I	0.4	3.8	0.1	3.8	0.7	1.2
Carbendazim	LC	CARB	F	0.6	4.0	0.5	4.0	0.1	1.0
Carbofuran	LC	CARB	I	0.1	3.8	0.03	3.8	0.4	1.0
Chlorothalonil	GC	ISOP	F	nd	nd	22.2	33.3	11.1	22.2
Chlorpyrifos	GC	OP	I	0.8	3.2	3.2	8.0	8.0	20.0
Chlorpyrifos-methyl	GC	OP	I	0.3	5.2	0.1	5.2	1.3	19.5
Clofentezine	LC	QUIN	A	1.0	3.9	1.0	3.9	9.7	48.6
Clothianidine	LC	NEO	I	0.9	10.6	0.3	4.3	1.4	17.0
Coumaphos	LC	OP	I	0.4	3.7	0.3	3.0	1.8	6.0
Cyfluthrin	GC	PYRE	I	12.3	61.5	12.3	30.8	76.9	230.7
Cyhalothrin-lambda	GC	PYRE	I	3.8	9.6	6.7	9.6	23.9	47.9
Cypermethrin	GC	PYRE	I	4.5	27.1	4.5	37.6	56.4	169.1
Cyproconazole	LC	TRIA	F	2.0	10.1	0.2	3.5	3.0	10.1
Deltamethrin	GC	PYRE	I	4.6	16.2	6.9	17.3	28.9	57.8
Diazinon	GC	OP	I	6.3	14.7	7.4	10.5	10.5	26.3
Dichloran	GC	OH	I	38.0	nd	19.0	57.0	47.5	nd
Dichlorvos	GC	OP	I	5.8	14.6	5.8	14.6	14.6	21.9
Dieldrin	GC	OH	I	3.9	9.8	3.9	29.5	9.8	24.6
Diethofencarbe	LC	CARB	F	0.2	3.8	0.04	3.8	0.6	1.9
Dimethoate	GC	OP	I	3.6	27.3	13.6	18.2	9.1	45.4
Endosulfan I	GC	OH	I	5.1	38.0	5.1	12.7	12.7	31.7
Endosulfan II	GC	OH	I	10.3	30.9	10.3	30.9	15.5	51.5
Endosulfan sulphate	GC	OH	I	5.1	8.4	1.2	3.4	8.4	21.1
Eprinomectin	LC	AVER	I	3.9	9.7	9.7	29.1	nd	nd
Esfenvalerate	GC	PYRE	I	10.1	30.2	10.1	30.2	25.1	150.9
Ethoprofos	GC	OH	I	0.6	3.6	1.3	6.4	3.2	13.7
Fenarimol	GC	CARBI	F	3.3	8.1	8.1	16.3	20.3	28.4
Fenitrothion	GC	OP	I	1.1	6.2	6.2	15.5	3.9	19.4
Fenoxycarbe	LC	CARB	I	0.6	4.1	0.1	4.1	1.0	3.3
Flusilazole	GC	TRIA	F	2.1	10.3	4.1	10.3	3.6	15.5
Hexachlorobenzene	GC	OH	F	0.8	3.9	0.2	3.9	9.7	24.3
Hexythiazox	LC	THIAZ	A	0.8	3.9	0.1	4.0	4.8	10.2
Imazalil	LC	IMI	F	1.4	10.2	0.7	4.1	6.9	25.5
Imidacloprid	LC	NEO	I	0.4	9.6	0.2	3.9	2.6	12.0
Iprodione	LC	DICA	F	9.7	19.5	9.7	19.5	15.6	48.7
Ivermectin	LC	AVER	I	11.7	23.5	23.5	70.4	nd	nd
Lindane	GC	OH	I	1.0	5.2	1.2	3.4	8.6	17.2
Malathion	GC	OP	I	7.8	15.6	5.5	11.7	39.1	58.6
Metamidophos	LC	OP	I	0.8	10.0	10.0	40.1	2.2	25.1
Methiocarbe	LC	CARB	M	0.4	10.3	0.01	4.1	0.2	0.5
Methomyl	LC	CARB	I	0.3	10.5	0.1	10.5	0.8	3.2
Methoxychlor	GC	OH	I	1.2	3.9	3.9	9.8	2.0	9.8
Moxidectin	LC	AVER	I	3.7	9.4	18.7	nd	nd	nd
Myclobutanil	GC	TRIA	F	10.7	21.4	10.7	32.2	10.7	37.5
o,p DDD	GC	OH	I	3.7	9.2	0.3	3.7	4.6	13.9
p,p-DDT	GC	OH	I	1.3	4.4	21.9	65.8	11.0	27.4
Paclobutrazol	GC	TRIA	F	4.3	10.8	7.5	16.2	3.8	10.8
Parathion	GC	OP	I	1.6	8.0	4.6	11.4	11.4	17.1
Penconazole	GC	TRIA	F	1.9	13.5	5.4	13.5	6.7	16.9
Permethrin	GC	PYRE	I	4.3	10.7	4.3	10.7	5.3	32.1
Phenthoate	GC	OP	I	0.6	14.4	0.3	14.4	1.4	14.4
Phosalone	GC	OP	I	4.1	10.2	4.1	10.2	10.2	15.4
Phosmet	GC	OP	I	9.8	19.7	3.9	9.8	14.8	24.6
Phoxim	LC	OP	I	1.8	7.3	0.1	7.3	2.7	15.5
Piperonyl Butoxyde	LC	BENZ	I	0.1	3.6	0.2	9.0	6.8	22.6
Prochloraz	LC	IMI	F	0.7	4.6	0.2	11.4	4.9	14.8
Procymidone	GC	DICA	F	nd	nd	1.3	3.7	nd	nd
Propargite	GC	SULES	A	11.9	34.1	17.1	25.6	42.7	128.0
Propiconazole	GC	TRIA	F	2.6	17.0	11.1	42.5	4.3	85.1
Pyriproxyfen	LC	PHENP	I	2.1	4.3	1.5	4.3	2.1	8.6
Tau-fluvalinate	GC	PYRE	I	3.7	9.1	3.7	9.1	4.6	22.8
Tebuconazole	GC	TRIA	F	5.1	17.9	12.8	25.6	12.8	38.4
Tetradifon	GC	OH	I	3.3	8.2	3.3	5.7	8.2	20.4
Thiamethoxam	LC	NEO	I	0.6	4.0	0.3	4.0	2.0	8.5
Thiophanate-methyl	LC	CARB	F	4.1	10.3	0.3	10.3	16.5	51.5
Tolclofos-methyl	GC	OP	I	0.3	3.0	0.1	3.0	1.1	11.4
Triadimenol	LC	TRIA	F	9.6	16.0	1.0	6.4	5.6	19.2
Triphenylphosphate	GC	OP	I	0.4	9.3	0.7	9.3	0.5	9.3
Vinclozoline	GC	DICA	F	4.0	10.1	4.0	10.1	1.5	12.6

1 Method: LC  =  liquid chromatography coupled with tandem mass spectrometry (LC−MS/MS); GC  =  gas chromatography coupled with Time of Flight mass spectrometry (GC-ToF).

2 Class: AVER  =  avermectine; BENZ  =  benzodioxole; CARB  =  carbamate; CARBI  =  carbinole; DICA  =  dicarboximide; FORM  =  formamidin; IMI  =  imidazole; ISOP  =  isophtalonitrile; NEO  =  neonicotinoid; OC  =  organochloride; OH  =  organohalogenus; OP  =  organophosphorus; PHENA  =  phenylamide; PHENP  =  phenylpyrazole; PYRE  =  pyrethroid; PYRI  =  pyrimidin; QUIN  =  quinoxaline; SULES  =  sulfite ester; THIAD  =  thiadiazin; THIAZ  =  thiazolidinone; TRIA  =  triazole.

3 Effect: A  =  acaricide; F  =  fungicide; I  =  insecticide; M  =  molluscicide; N  =  nematodicide.

4 LOD  =  limit of detection in ng/g; nd  =  not determined.

5 LOQ  =  limit of quantification in ng/g; nd  =  not determined.

### Statistical analyses

The statistical parameters for the concentrations (mean, median and standard deviation) were calculated from all the analyzed samples of each matrix and not only from samples for which the residues were detected or quantified. When a compound was not detected (< LOD), the concentration used for statistical analysis was ½ LOD [35]. When a compound was not quantified (> LOD and < LOQ), the concentration used was ½ (LOD + LOQ).

For each matrix, statistical analyses were performed only for residues that were detected or quantified at least once. We transformed the data into a present/absent dataset and considered the number of compounds detected and/or quantified in each sample as an explicative variable in the following models.

Linear mixed effects models were used to perform a comparison between the number of residues detected or quantified (i) in honey bees (n = 141), honey (n = 141) and pollen (n = 128); (ii) in different landscape structures (rural-grassland, rural-cultivated, urban and island); and (iii) for different sampling periods (AM8, JJ8, JA8, SO8, AM9, JJ9, JA9 and SO9). These models were the best way to take into account the repeated measurements on each apiary. The linear mixed effect models are the theoretical presentation of ANOVA for repeated measurements.

For each of these three models, the assumption of independence and normality of the residues and random effects was checked through diagnostic graphs generated by the parametric estimation theory of mixed effects models (data not shown) [36]. Then, Tukey post-hoc tests (a specific version designed for mixed effects models) were used to implement multiple comparisons of the means in each model, (i) difference in matrix, (ii) difference in landscape and (iii) difference in sampling periods.

The statistical analyses were performed using R software with the “nlme package” for the mixed effects and the “multcomp package” for the post-hoc tests [37]. Significant differences were evaluated based on a 5% type one error (α = 5%).

## Results

### Multiple contaminant residues were detected in the honey bee, honey and pollen matrices

Among the 141 honey bee, 141 honey and 128 pollen samples collected from 18 apiaries during 2008 and 2009, 102 (72.3%), 135 (95.7%) and 75 (58.6%) of the samples, respectively, were contaminated by at least one contaminant.

Twenty compounds were detected in honey bees (**Table 2**), with up to 6 different residues in a single sample and a mean of 1.4 residues per analyzed sample. In this matrix, 36.2% of the samples contained at least 2 residues, and 18.4% contained at least 3 residues. Twenty-eight compounds were detected in honey (**Table 3**), with up to 8 different residues in a single sample and a mean of 2.9 residues per analyzed sample. In this matrix, 80.8% of the samples contained at least 2 residues, and more than 3 residues were detected in 57.4% of these samples. Twenty-three compounds were detected in pollen (**Table 4**), with up to 7 different residues in a single sample and a mean of 1.1 residues per analyzed sample. In this matrix, 30.5% of the samples contained at least 2 residues, and 11.7% of the samples contained at least 3 residues. The mixed effects models and the Tukey post-hoc tests indicated a significant difference between the number of residues in the honey and honey bee samples (Tukey test, z = 9.991, p<0.0001) and between the number of residues in the honey and pollen samples (Tukey test, z = −11.578, p<0.0001).

**Table 2:** Summary of contaminant residues detections in honey bee samples from western France honey bee colonies.

Compound	Class^1^	Effect^2^	%^3^	Detections				
				Min^4^	Max^4^	Mean^5^	Median^5^	SD^5^
Amitraz I	FORM	A	5.0	> LOD and < LOQ	29.60	9.99	9.25	3.27
Amitraz II	FORM	A	16.3	> LOD and < LOQ	17.00	3.07	2.15	2.41
Benalaxyl	PHENA	F	1.4	> LOD	< LOQ	3.05	2.85	1.69
Carbaryl	CARB	I	2.1	> LOD	< LOQ	0.24	0.20	0.28
Carbendazim	CARB	F	41.1	> LOD and < LOQ	66.30	2.04	0.30	6.59
Chlorpyrifos	OP	I	3.5	> LOD and < LOQ	180.20	1.72	0.40	15.14
Coumaphos	OP	A	17.8	> LOD and < LOQ	47.30	1.04	0.20	4.33
Cypermethrin	PYRE	I	1.4	28.50	48.80	2.52	2.00	4.52
Diazinon	OP	I	0.7	> LOD	< LOQ	3.20	3.15	0.62
Fenoxycarb	CARB	I	0.7	20.10	20.10	0.44	0.30	1.67
Flusilazole	TRIA	F	1.4	> LOD	< LOQ	1.12	1.05	0.61
Hexythiazox	THIAZ	A	0.7	> LOD	< LOQ	0.41	0.40	0.16
Phosalone	OP	I	0.7	> LOD	< LOQ	2.09	2.05	0.43
Phosmet	OP	I	2.8	> LOD and < LOQ	62.20	5.52	4.90	5.01
Piperonyl Butoxide	BENZ	I	2.1	> LOD	< LOQ	0.09	0.05	0.26
Propiconazole	TRIA	F	1.4	> LOD and < LOQ	7.80	0.37	0.30	0.65
Pyriproxyfen	PHENP	I	1.4	> LOD	< LOQ	1.08	1.05	0.26
Tau-fluvalinate	PYRE	I	7.1	> LOD and < LOQ	52.90	3.41	1.85	7.20
Thiophanate-methyl	CARB	F	5.7	> LOD and < LOQ	2418.70	22.96	2.05	207.54
Triphenylphosphate	OP	I	24.8	> LOD and < LOQ	61.60	1.95	0.20	5.86

1 Class: BENZ  =  benzodioxole; CARB  =  carbamate; FORM  =  formamidin; OP  =  organophosphorus; PHENA  =  phenylamide; PHENP  =  phenylpyrazole; PYRE  =  pyrethroid; THIAZ  =  thiazolidinone; TRIA  =  triazole.

2 Effect: A  =  acaricide; F  =  fungicide; I  =  insecticide.

3 n = 141 honey bee samples.

4 Min  =  minimum in ng/g; LOD  =  limit of detection; LOQ  =  limit of quantification.

5 Mean, Median and SD (standard deviation) were calculated taking into account all the analyzed samples.

**Table 3:** Summary of contaminant residues detections in honey samples from western France honey bee colonies.

Compound		Class^1^	Effect^2^	%^3^	Detections				
					Min^4^	Max^4^	Mean^5^	Median^5^	SD^5^
Amitraz I	FORM		A	4.2	> LOD and < LOQ	26.00	5.65	5.00	3.29
Amitraz II	FORM		A	68.8	> LOD and < LOQ	116.10	10.21	2.30	18.62
Bupirimate	PYRI		F	1.4	> LOD	< LOQ	2.95	2.85	0.84
Buprofezin	THIAD		I	1.4	> LOD and < LOQ	42.80	12.30	11.95	3.00
Carbaryl	CARB		I	6.4	> LOD and < LOQ	4.10	0.18	0.05	0.53
Carbendazim	CARB		F	64.5	> LOD and < LOQ	87.90	2.89	2.25	8.42
Carbofuran	CARB		I	2.1	> LOD	< LOQ	0.06	0.02	0.28
Chlorpyrifos-methyl	OP		I	1.4	> LOD	< LOQ	0.24	0.20	0.31
Coumaphos	OP		A	78.0	> LOD and < LOQ	56.40	2.48	1.65	5.69
Cypermethrin	PYRE		I	0.7	> LOD	< LOQ	2.38	2.25	1.58
Cyprocanozole	TRIA		F	11.3	> LOD and < LOQ	3.80	0.31	0.10	0.62
Diazinon	OP		I	2.1	> LOD and < LOQ	14.00	3.85	3.70	1.06
Diethofencarb	CARB		F	0.7	> LOD	< LOQ	0.03	0.02	0.16
Endosulfan-beta	OH		I	0.7	> LOD	< LOQ	5.26	5.15	1.30
Fenoxycarb	CARB		I	0.7	> LOD	< LOQ	0.06	0.05	0.17
Flusilazole	TRIA		F	2.1	> LOD	< LOQ	2.16	2.05	0.75
Hexythiazox	THIAZ		A	1.4	> LOD	< LOQ	0.08	0.05	0.24
Imazalil	IMI		F	4.2	> LOD	< LOQ	0.44	0.35	0.42
Imidacloprid	NEO		I	2.1	> LOD	< LOQ	0.14	0.10	0.28
Phosmet	OP		I	12.8	> LOD and < LOQ	42.10	3.57	1.95	5.07
Phoxim	OP		I	2.1	> LOD	< LOQ	0.13	0.05	0.53
Piperonyl Butoxide	BENZ		I	8.5	> LOD	< LOQ	0.48	0.10	1.26
Prochloraz	IMI		F	1.4	> LOD	< LOQ	0.18	0.10	0.68
Pyriproxyfen	PHENP		I	3.5	> LOD	< LOQ	0.83	0.75	0.40
Tau-fluvalinate	PYRE		I	5.0	> LOD and < LOQ	30.00	2.30	1.85	2.73
Tebuconazole	TRIA		F	0.7	> LOD	< LOQ	6.49	6.40	1.08
Thiophanate-methyl	CARB		F	1.4	4.00	5.30	0.21	0.15	0.54
Triphenylphosphate	OP		I	2.1	> LOD	< LOQ	0.45	0.35	0.67

1 Class: BENZ  =  benzodioxole; CARB  =  carbamate; FORM  =  formamidin; IMI  =  imidazole; NEO  =  neonicotinoid; OH  =  organohalogenus; OP  =  organophosphorus; PHENP  =  phenylpyrazole; PYRE  =  pyrethroid; PYRI  =  pyrimidin; THIAD  =  thiadiazin; THIAZ  =  thiazolidinone; TRIA  =  triazole.

2 Effect: A  =  acaricide; F  =  fungicide; I  =  insecticide.

3 n = 141 honey samples.

4 Min  =  minimum in ng/g; LOD  =  limit of detection; LOQ  =  limit of quantification.

5 Mean, Median and SD (standard deviation) were calculated taking into account all the analyzed samples.

**Table 4:** Summary of contaminant residues detections in pollen samples from western France honey bee colonies.

Compound	Class^1^	Effect^2^	%^3^	Detections				
				Min^4^	Max^4^	Mean^5^	Median^5^	SD^5^
Amitraz I	FORM	A	1.6	> LOD and < LOQ	115.20	24.14	23.15	8.67
Amitraz II	FORM	A	14.8	> LOD and < LOQ	129.40	7.39	4.10	13.82
Bupirimate	PYRI	F	0.8	> LOD	< LOQ	1.48	1.40	0.95
Carbaryl	CARB	I	7.8	> LOD and < LOQ	14.67	0.70	0.35	1.68
Carbendazim	CARB	F	34.4	> LOD and < LOQ	2595.00	24.31	0.05	229.47
Carbofuran	CARB	I	1.6	> LOD and < LOQ	2.30	0.22	0.20	0.19
Chlorpyrifos	OP	I	3.9	> LOD and < LOQ	139.50	5.61	4.00	12.53
Coumaphos	OP	A	3.9	> LOD and < LOQ	40.40	1.95	0.90	5.10
Cyprocanozole	TRIA	F	0.8	22.30	22.30	1.66	1.50	1.84
Dieldrin	OH	I	0.8	> LOD	< LOQ	5.0	4.90	1.09
Diethofencarb	CARB	F	0.8	2.60	2.60	0.32	0.30	0.32
Dimethoate	OP	I	0.8	> LOD	< LOQ	4.73	4.55	2.01
Flusilazole	TRIA	F	2.3	19.90	51.60	2.60	1.80	5.53
Imidacloprid	NEO	I	0.8	> LOD	< LOQ	1.35	1.30	0.53
Iprodione	DICA	F	0.8	> LOD	< LOQ	7.99	7.80	2.15
Phosmet	OP	I	7.4	> LOD and < LOQ	78.10	9.38	7.40	8.80
Piperonyl Butoxide	BENZ	I	0.8	> LOD	< LOQ	3.49	3.40	1.00
Pyriproxyfen	PHENP	I	4.7	> LOD	< LOQ	5.85	5.35	2.28
Tau-fluvalinate	PYRE	I	3.1	> LOD and < LOQ	85.42	3.52	2.30	8.31
Thiophanate-methyl	CARB	F	1.6	1395.00	3674.00	47.72	8.25	345.52
Triadimenol	TRIA	F	2.3	34.30	35.70	8.64	8.00	4.12
Triphenylphosphate	OP	I	9.4	> LOD	< LOQ	0.69	0.25	1.36
Vinclozolin	DICA	F	0.8	70.31	70.31	1.29	0.75	6.15

1 Class: BENZ  =  benzodioxole; CARB  =  carbamate; DICA  =  dicarboximide; FORM  =  formamidin; NEO  =  neonicotinoid; OH  =  organohalogenus; OP  =  organophosphorus; PHENP  =  phenylpyrazole; PYRE  =  pyrethroid; PYRI  =  pyrimidin; TRIA  =  triazole.

2 Effect: A  =  acaricide; F  =  fungicide; I  =  insecticide.

3 n = 128 honey bee samples.

4 Min  =  minimum in ng/g; LOD  =  limit of detection; LOQ  =  limit of quantification.

5 Mean, Median and SD (standard deviation) were calculated taking into account all the analyzed samples.

A total of 37 different compounds were detected when considering all the matrices (**Tables 2** **,** **3** **and** **4**), and only 12 compounds were detected in all three matrices: 3 acaricides (2 metabolites of amitraz, amitraz I and amitraz II, and coumaphos), 3 fungicides (carbendazim, flusilazole and thiophanate-methyl) and 6 insecticides (carbaryl, phosmet, piperonyl butoxide, pyriproxyfen, tau-fluvalinate and triphenylphosphate).

### Most prevalent detected contaminant residues and their concentrations in the three beehive matrices

The most frequently detected residues (in more than 10% of the samples) were 1) carbendazim (41.1%), triphenylphosphate (24.8%), coumaphos (17.8%) and amitraz II (16.3%) in honey bees (**Table 2**); 2) coumaphos (78.0%), amitraz II (68.8%), carbendazim (64.5%), phosmet (12.8%) and cyproconazole (11.3%) in honey (**Table 3**), and 3) carbendazim (34.4%) and amitraz II (14.8%) in pollen (**Table 4**).

The highest maximum concentrations were obtained in pollen for the fungicides thiophanate-methyl (max  = 3674.00 ng/g) and carbendazim (max  = 2595.00 ng/g). Thiophanate-methyl (max  = 2418.70 ng/g) and the insecticide chlorpyrifos (max  =  180.20 ng/g) were also quantified in high concentrations in honey bees, although they were rarely detected (5.7% and 3.5% for thiophanate-methyl and chlorpyrifos, respectively). In honey, the highest concentrations concerned the acaricide amitraz II (max  = 116.10 ng/g) and the fungicide carbendazim (max  =  87.90 ng/g). Although coumaphos was more frequently found in honey (78% of samples) compared with honey bees (17.8%) and pollen (3.9%), the maximal concentrations did not differ significantly between the three matrices (56.40, 47.30 and 40.40 ng/g).

The neonicotinoid imidacloprid was only detected in 3/141 honey samples (2.1%) and in 1/128 pollen samples (0.8%) and was not found among the 141 honey bee samples. Pyrethroids were also rarely detected, with tau-fluvalinate as the most frequent (7.1% in bees, 5.0% in honey and 3.1% in pollen) followed by cypermethrin (1.4% in bees and 0.7% in honey). In contrast, bifenthrin, cyfluthrin, deltamethrin, esfenvalerate, lambda cyhalothrin, permethrin and tefluthrin were never detected.

Honey was the matrix most contaminated by triazole fungicides, with 5 different compounds, including cyproconazole, which was detected in 11.3% of the samples. The azole fungicide concentrations were the highest in pollen and were always higher than the LOQ. Three samples were contaminated by flusilazole (51.60, 35.88 and 19.9 ng/g), three samples by triadimenol (35.70, 35.4 and 34.3 ng/g), and one sample by cyproconazole (22.30 ng/g).

Coumaphos (17.8% in bees, 78.0% in honey and 3.9% in pollen), triphenylphosphate (24.8% in bees, 2.1% in honey and 9.4% in pollen) and phosmet (2.8% in bees, 12.8% in honey and 7.4% in pollen) were the three most prevalent cholinesterase inhibitor insecticides detected. The insecticides carbaryl and chlorpyrifos were rarely observed. Carbaryl was detected in 3/141, 9/141 and 10/128 of the honey bee, honey and pollen samples, respectively. Chlorpyrifos was detected in 5/141 honey bee samples and in 5/128 pollen samples, but the maximum concentrations were very high (180.20 ng/g and 139.50 ng/g in the honey bee and pollen samples, respectively). The apicultural matrices were less contaminated by other carbamate and organophosphorus compounds, such as carbofuran, diazinon, dimethoate, fenoxycarb and phoxim, with detection rates between 0% and 2.1% depending on the matrices and compounds.

### Contamination according to the landscape context and the sampling period

**Figure 2:**
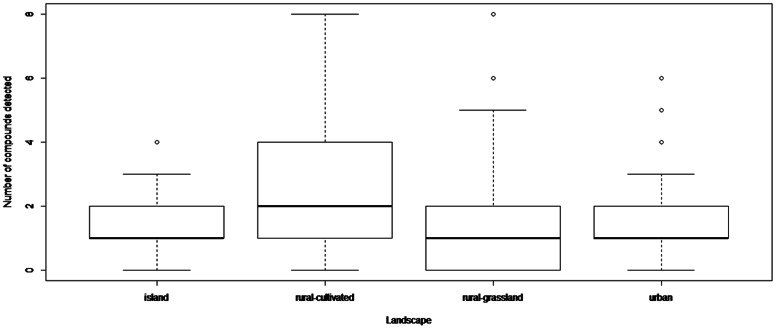
Number of compounds detected according to the landscape context. The number of compounds was calculated irrespective of the matrix (honey bees, honey and pollen) for each landscape context (rural-grassland, rural-cultivated, island and urban landscapes).

**Figure 2** shows the number of agricultural and veterinary residues (all matrices confounded) detected according to the landscape context. Even if the median for rural-cultivated landscape appears to be higher than the others, the linear mixed effects models failed to reveal a significant difference. This was due to a high standard error of the different estimations (Tukey test, −2.3<z<2.3, p>0.05).

**Figure 3:**
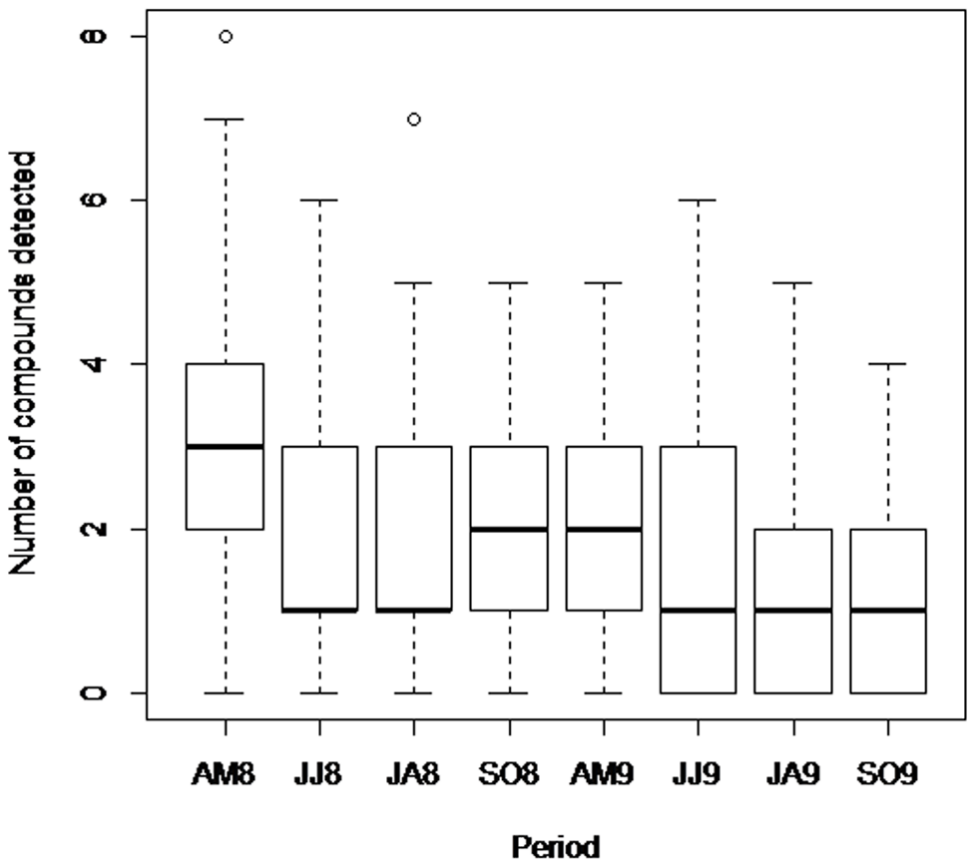
Number of compounds detected according to the period. The number of compounds was calculated irrespective of the matrix (honey bees, honey and pollen) for each period: late April-early May 2008 (period AM8) and 2009 (period AM9), late June-early July 2008 (period JJ8) and 2009 (period JJ9), late July-early August 2008 (period JA8) and 2009 (period JA9), and late September-early October 2008 (period SO8) and 2009 (period SO9).

**Figure 3** shows the number of agricultural and veterinary residues (three matrices confounded) detected during 8 periods over 2008 and 2009. Contamination was higher during the period AM8 (late April-early May 2008) than during all other periods (Tukey test, -z<−3.68, p<0.01).

## Discussion

### Most prevalent contaminants in beehive matrices

The fungicide carbendazim and the acaricides amitraz and coumaphos, which are commonly used in beehives to control the parasitic mite *Varroa destructor*, were the three most prevalent residues. Several other studies have previously demonstrated that the chemicals used by beekeepers inside the hives are frequently found in the apicultural matrices [30], . Amitraz residues (amitraz I and amitraz II) were mainly detected in bees at the beginning of autumn (periods SO8 and SO9), which corresponds to periods of treatments against *Varroa destructor*. In the pollen matrix, this acaricide was detected during the periods AM8, SO8, AM9 and SO9, which correspond to the end of the beekeeping season with anti-*Varroa* treatments and the beginning of the next beekeeping season. The presence of amitraz in the pollen matrix might be the result of transfer from contaminated bees because its use as a plant-protective acaricide is no longer authorized in France. Surprisingly, amitraz residues were identified in honey samples for all the eight periods. In contrast, Maver and Poklukar [41] and Martel et al. [42] did not detect any amitraz residue in honey after treatment with this compound, which may be explained by their LOD and LOQ being at least ten-fold higher than ours. Coumaphos, another acaricide extensively used against *Varroa* in recent decades, was also frequently detected in apicultural matrices [28], [30]. In addition, many studies indicate that coumaphos was persistent in wax and diffused from wax to honey in high proportions [43]−[44]. Although this acaricide is now banned in France, we detected this molecule in most honey samples (110/141 samples). This might be the result of past chronic use, accumulation and then transfer from contaminated wax, except for one apiary, where an illegal use of coumaphos was demonstrated after an investigation.

The high prevalence of the fungicide carbendazim in the three beehive matrices might result from its very wide use on orchards, vineyards, and grains, along with oleaginous and vegetable crops, both in agricultural and in gardening treatments. Otherwise, carbendazim is also a metabolite of thiophanate-methyl, and some detection of this substance might be linked to the field use of thiophanate-methyl. This is most likely the reason why all samples contaminated by thiophanate-methyl also contained carbendazim residues.

Among the five most frequently detected residues, Mullin et al. [30] identified two in-hive miticides, coumaphos and amitraz, and the fungicide chlorothalonil, widely applied for the control of fungal diseases in agricultural and gardening treatments [45]. Those results were consistent with those of the present study, even if the fungicide was not the same, which was most likely due to different agricultural treatment practices. The acaricide fluvalinate, which was the most frequent residue found by Mullin et al. [30], was detected in lower frequency in the present study and in a recent French study [31], reflecting different apicultural practices in Europe and North America. Systematic combinations of acaricides used to fight *Varroa* or fungicides and other pesticides have been reported in many studies [30], [46], and the interaction between such compounds can induce lethal or adverse sublethal effects in honey bees [17], .

### Contamination according to the landscape context and to the sampling period

The spatial patterns of the contamination of beehive matrices by environmental pollutants such as polycyclic aromatic hydrocarbons or heavy metals have been previously studied [26], [32−33], . Most of these studies compared the contamination in both urban and wild sites. To our knowledge, the pesticide spatial distribution in bees, honey or pollen was only studied in different areas within the same landscape context and, in particular, cultivated landscapes [28−29], [31], . The present study is the first to monitor beehive matrix contamination by pesticides in 4 different landscape structures characterized by different pesticide and veterinary drug use patterns (private or professional uses, veterinary, apicultural or agricultural uses). Apiaries in rural-cultivated landscapes were more contaminated than apiaries in all other landscape types, but the differences were not significant.

Acaricide residues were observed in all the 18 apiaries, and the differences in spatial distribution were linked to the nature of the compounds used in agricultural treatments such as organophosphorus and carbamate insecticides and azole fungicides. In rural-cultivated landscapes (dominated by permanent, oleaginous, grain crops, market gardening), agricultural treatments are performed on large plots and often on melliferous plants (*Asteraceae*, *Brassicaceae*, *Fabaceae*). These treatments are more likely to contaminate honey bees than local treatments in an urban context, particularly if the urban district green services practice a zero pesticide policy. Despite this trend, the present results indicate that some apiaries located in urban landscapes, which are supposedly less exposed to pesticide pressure, were more contaminated than apiaries in rural landscapes.

Previous studies have demonstrated seasonal variation in beehive matrix contamination by polycyclic aromatic hydrocarbons [33] and heavy metals [26], [32], [51]. The contamination levels were linked to the meteorological conditions and were generally higher during the dry months. However, few studies have reported the evolution of pesticide contamination throughout the beekeeping season. In the present study, the most contaminated period corresponded to late April-early May in 2008 and was associated with agricultural uses and crop treatments [54] and with a high foraging activity. Ghini et al. [53] demonstrated that the maximum level and frequency of pesticides (organophosphate and carbamate residues) occurred in the late spring (May and June). Samplings of the present study were collected 4 times per year and reflected a single time point and not the contamination kinetics throughout the year. This sampling methodology might explain the differences with Ghini et al. [53], who performed samplings each month from April to October 2000. The higher contamination in spring 2008 was not observed in 2009, most likely due to differences in the meteorological conditions and treatment time.

### Pesticide and veterinary drug contamination of beehive matrices

Honey was the most contaminated matrix in the present study when taking into account the number of residues observed by matrix and by sample. These results are not in agreement with the results of other studies in which pollen was observed to be the most contaminated matrix [30]−[31]. Indeed, Mullin et al. [30] demonstrated that among 140 honey bee and 350 pollen samples collected in the USA, in both 2007 and 2008, 91.4% and 99.1% of the samples were contaminated, respectively. However, the context of their study and the method used for pesticide detection and quantification were different: (i), samples were collected to investigate possible threats to colony health for the CCD (Colony Collapse Disorder) working group; (ii), more than twice as many compounds were searched, with an average of 171 pesticides and toxic metabolites studied per analysis; and (iii), the honey matrix was not analyzed for pesticide presence. Another recently published study on beehive matrices collected in France between 2002 and 2005 also demonstrated that pollen samples were the most contaminated (69.5%) compared with honey bee (44.3%) and honey (43.1%) samples [31]. Whereas LOD and LOQ were similar for the different matrices in these two previous studies [30]−[31], they were generally higher in pollen than in the honey bee or honey matrices in our survey. This could explain the different prevalence for residues among our matrices, and in particular, the lower frequency of detection in pollen samples. However, for most compounds, the maximum concentration and the mean concentration were higher in pollen than in honey bees and honey. If a lower LOD and LOQ could be achieved for pollen samples, it is likely that pollen would be the best matrix for assessing the presence of pesticides in foraging areas.

### Other contaminants and implications for bee colony health

Systemic insecticides, including neonicotinoids, have been demonstrated to present a high acute and chronic toxicity in bees [16]–[17], [55]–[59]. A survey in France from 2002 to 2005 indicated that imidacloprid was frequently detected in honey bee matrices: 11.2%, 40.5% and 21.8% of the honey bee, pollen and honey samples were found to be contaminated, respectively [31]. In contrast, this molecule was rarely detected in our study. A potential explanation is that our data were from a more recent field-study and might reflect changes in agricultural practices; for example, imidacloprid is being gradually replaced by new neonicotinoids such as thiamethoxam [60], even if this neonicotinoid was never detected in the present samples. The low presence of neonicotinoids in our survey was most likely linked to the multi-residue analysis, which is characterized by a high LOD. Indeed, these molecules are used at very low doses, and our detection method was not sensitive enough to detect them in most samples. However, no adverse effect has been established by previous studies at our concentrations and at other field-relevant doses for pollen and nectar.

Like neonicotinoids, pyrethroids can induce adverse acute sublethal effects in bees [16]–[17]. However, except for tau-fluvalinate and cypermethrin, other pyrethroids were never detected in our study. These contamination levels in the present study were consistent with those of Chauzat et al. [31] but were much lower than those of Mullin et al. [30], in relation to lower LODs than ours for these residues. In addition, it has been demonstrated that a synergistic action between pyrethroids and azole fungicides can occur [61] and may increase the risk to bees. Interestingly, one apiary located in a rural-cultivated landscape displayed pyrethroid (tau-fluvalinate) and azole fungicide (flusilazole) contamination in the same period and in both pollen and bee matrices. Important levels of bee mortality were noticed in this apiary during and after the sampling, but no direct relationship with the co-contamination by tau-fluvalinate and flusilazole was demonstrated.

Carbaryl was frequently detected in pollen and honey samples. Ninety percent of carbaryl detections concerned the samples collected in 2008, which is consistent with the ban on the use of this compound in November 2008 in France, and confirmed that the beehive matrices are very sensitive in terms of reflecting agricultural practices and pesticide use. In addition, it was previously demonstrated that sublethal exposure to carbaryl has adverse effects on the longevity and foraging activities of honey bees [62]. Although the concentrations determined in the present study were lower than those in this semi-field study, chronic exposure to this insecticide might cause problems to bee colonies.

Except for coumaphos, phosmet and triphenylphosphate, few organophosphorus compounds were detected, which is contrast to the results of other studies. For example, Ghini et al. [53] found that twelve organophosphorus compounds were present in more than 10% of examined samples (58% for malathion and 53% for fenitrothion). The adverse effects of these compounds have been demonstrated, and their use is now controlled and limited. When considering the weight of one bee (0.1108 g, mean weight determined for the sampled honey bees in the present study), some maximum concentrations in the honey bees were very high and close to or above the LD_50_ (lethal dose 50) divided by the safety factor of 100 that is regularly used in environmental toxicology. Such was the case for the insecticides chlorpyrifos (LD_50_  = 122 ng/bee, maximum concentration determined  =  19.97 ng/bee) and phosmet (LD_50_  = 803 ng/bee, maximum concentration determined  = 6.89 ng/bee). Such concentration levels might have affected the bee health in the corresponding samples either by inducing direct mortality or via sublethal effects on bee physiology and behavior [16]. The maximum concentrations in pollen were similar to those measured in bees for chlorpyrifos and phosmet, and the sublethal effects of the residues in bees, especially in brood, were likely the result of delivery through consumption of contaminated pollen samples. The organophosphate triphenylphosphate is not allowed for agricultural use in France, but it is highly used in industrial settings as a flame retardant [63]. Thus, this ubiquitous environmental pollutant might contaminate apicultural matrices via atmospheric residues. Honey bees are more likely to be exposed to atmospheric pollutants during their foraging due to their flight, their hairy bodies and their ability to hold residues, which might explain the contamination levels found in the present study.

Because of the difference in residue LOD and LOQ for each matrix, no correlation was tested between the three beehive matrices collected from the same apiary and at the same period. Such differences might explain the fact that a residue found in one matrix was not systematically present in the two others. Other explanations might include the following features: 1) the sampling methodology at a single time point (honey was stored inside the hive for several days before the sampling, pollen was collected from a pollen trap set up 3 days before the sampling, and foraging honey bees were directly collected on the hive's flight board), 2) the specific biotransformations of residues in each matrix, and 3) the ability of each matrix to reflect contamination in time.

In conclusion, our field study revealed the widespread presence of multiple residues in honey, honey bees and pollen with different distribution patterns according to the landscape context and the sampling period. This contamination by multiple residues also raises the issue of the impact of the combinations of these pesticides and veterinary drugs and their potential synergistic effects on the health of bees and other pollinators [64], [65].
